# Risk Communication and Community Engagement During the Migrant Worker COVID-19 Outbreak in Singapore

**DOI:** 10.1177/10755470211061513

**Published:** 2022-04

**Authors:** Wai Jia Tam, Nina Gobat, Divya Hemavathi, Dale Fisher

**Affiliations:** 1 National University of Singapore, Singapore; 2 University of Oxford, UK; 3 National University Hospital, Singapore

**Keywords:** migrant workers, risk communication, community engagement, COVID-19, Singapore

## Abstract

In early phases of the COVID-19 pandemic in Singapore, Risk Communication and Community Engagement (RCCE) with large, diverse communities of migrant workers living in high-density accommodation was slow to develop. By August 2020, Singapore had reported 55,661 cases of COVID-19, with migrant workers comprising 94.6% of the cases. A system of RCCE among migrant worker communities in Singapore was developed to maximize synergy in RCCE. Proactive stakeholder engagement and participatory approaches with affected communities were key to effective dissemination of scientific information about COVID-19 and its prevention.

## Introduction

Risk communication and community engagement (RCCE) are essential components of a broader health emergency preparedness and response action plan (Pan American Health Organization, 2020). It describes two distinct but interrelated approaches to supporting communities to adopt disease-safe behaviors and take community action in support of ending disease transmission (Bedson et al., 2020). Risk communication is the multidirectional communication and engagement with affected populations so that they can make informed decisions to protect themselves (Pan American Health Organization, 2020). In the context of the COVID-19 pandemic, it includes effective dissemination of scientific information and also the range of communication actions required through the preparedness, response, and recovery phases, to encourage positive behavior change, and the maintenance of trust (Pan American Health Organization, 2020). Community engagement is a critical component of civil society, international development practice, and humanitarian assistance and is based on the premise that communities should be listened to and have a meaningful role in processes and issues that affect them (United Nations Children’s Fund, 2020). The global strategy outlines how RCCE should be community-centered, trust-nurturing, data-informed (United Nations Children’s Fund et al., 2020). This article describes how a system of RCCE was developed from a ground-up approach into a sustainable, coordinated, nationwide effort with effective strategies for scientific communication to large, diverse communities of migrant workers.

## Local Setting

Migrant workers comprise 24.3% of Singapore’s population (Yi et al., 2020). Male “Work Permit” holders, mostly aged 18 to 50 years old, number 716,200 and originate from mainly Bangladesh, India, and China, working in construction, manufacturing, marine, or cleaning industries (Ministry of Health, 2020). Approximately 323,000 migrant workers reside in one of 43 purpose-built dormitories, which are barracks-style and apartment-style residential buildings, accommodating up to 25,000 residents, housing six to 32 residents per unit (Chia et al., 2020; Ministry of Manpower, 2021c, 2021d; Urban Redevelopment Authority, 2021; Zhuo, 2020). Migrant workers fall outside the universal health coverage system and jurisdiction of local labor laws with regard to minimum wage, employment mobility, and occupational rights such as rest days or vacation (Cheong, 2020; Humanitarian Organization for Migration Economics, 2020; Rajaraman et al., 2020). In this article, “Migrant Worker” refers to male Work Permit holders (Gorny et al., 2020).

In January 2020, Singapore first identified a person with COVID-19 infection (Ministry of Manpower, 2021d). COVID-19 spread widely among migrant workers in dormitories, and all dormitories were locked down in April 2020 (Ministry of Manpower, 2021e; Rajaraman, 2020). By August 2020, Singapore had reported 55,661 laboratory-confirmed cases of COVID-19, where migrant workers comprised 94.6% of the cases (Chia et al., 2020; Ministry of Manpower, 2021b). From April to August 2020, Singapore implemented large-scale institutional isolation units called community care facilities (CCFs) for COVID-19-positive migrant workers (Phua, 2020). The three regional health clusters in Singapore’s public health care system operated these facilities and conducted swab and serology operations at dormitories (Phua, 2020).

At the onset of the pandemic, RCCE activities among the large, diverse communities of migrant workers living in high-density accommodation were poorly coordinated and were fronted by government authorities and nonprofit organizations (Yi et al., 2020). Early strategies from the multiministry Joint Task Force, such as placement of migrant workers based on the results of extensive systematic testing regimens, were communicated with difficulty due to language barriers and lack of communication resources (Gorny et al., 2020; Rajaraman et al., 2020).

## Approach

**Figure 1.**
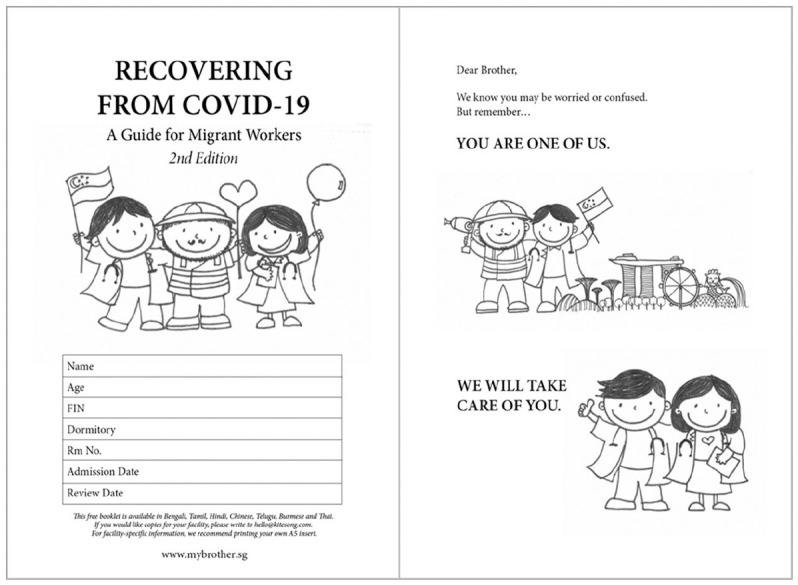
An example of illustrations in the health resources created, which included characters that were friendly, relatable, and culturally sensitive, drawing elements from their daily lives to ensure contextualization and relatability.

In May 2020, health workers at CCFs formed an informal cross-cluster network to pool multilingual resources to address communication challenges with migrant worker patients. This early attempt to coordinate resources was ad hoc. Volunteer doctors developed a pictorial, multilingual health booklet to orientate incoming patients that was based on contextual realities of migrant worker living conditions, workers’ feedback and best available information (see Figure 1). The urgency of this initial request prevented formal intervention development work. Leveraging on inherent hierarchy structures, migrant worker leaders collected feedback on behalf of the emerging RCCE team. Individuals from health clusters, nonprofit organizations, and government authorities connected via text messaging and email groups through informal networks to order the booklets and formed the first RCCE working group, which networked strategically to discuss future plans. The nonpartisan branding of the booklet was crucial to its wide uptake by high-level stakeholders, as it conveyed inclusivity. Multimodal resources comprising health booklets, posters, face-to-face engagements, podcasts, webinars, and social media activities were co-developed with workers to share health messages. Early, proactive stakeholder engagement encouraged ownership and broad dissemination. To scale RCCE efforts, a local steering committee was created, supported by an international technical advisory group. Staff were recruited to organize volunteers, manage donor funding, and implement programs.

The uncontrolled spread of COVID-19 among migrant worker communities meant needing to rapidly, responsively deliver RCCE activities. The RCCE program content and structure was shaped through the following activities: (a) conducting a Strengths, Weaknesses, Opportunities, Threats (SWOT) analysis; (b) undertaking knowledge, attitudes, practices (KAP) surveys to establish baselines; (c) curating content (including co-development with migrant workers, piloting, collecting feedback); (d) mapping media consumption channels; and (e) establishing multiple distribution channels. Challenges and solutions to these are detailed in Table 1.

**Table 1.** Key Challenges and Bottom-Up Solutions to Delivering RCCE Among a Large Migrant Worker Population in Singapore During the COVID-19 Pandemic.

Challenges	Opportunities and solutions
Stakeholders	
Poor coordination between nonprofit organizations and health clusters, with no central leadership.	An RCCE working group was established, with regular meetings to strategize plans for coordination as a network.
Limited stakeholder buy-in and support.	Proactive identification and engagement of high-level leaders and stakeholders was done, including at policy-making level for strategic planning even after acute crisis phase. There was early and broad sharing of tools and products.
RCCE was not prioritized as a key pillar of outbreak response due to being misunderstood as workers’ welfare.	Influential high-level leadership addressed skepticism toward RCCE proactively.
The local steering committee comprised only of doctors initially.	Partners from diverse backgrounds were included to leverage on more strengths and facilitate cross-disciplinary collaborations. Policymakers from government ministries were engaged to provide input and receive on-ground feedback.
Migrant Workers	
Migrant workers in Singapore are culturally diverse and speak various languages.	Volunteers who spoke in the various eight languages were recruited to assist with efforts.
Lack of a centralized channel to receive health messaging in the early parts of the outbreak.	Stronger communication channels were built by utilizing commonly used social media sites, and partnerships with influential migrant worker personalities and government authorities.
Lack of understanding on migrant workers’ access to health information.	Continuous adoption of innovative approaches to engage with migrant workers, both online and offline was done.
Migrant workers were reluctant to seek medical attention at times.	Podcasts, videos, and resources were produced by migrant worker leaders to encourage seeking medical attention early when needed.
Challenges (e.g., movement restrictions, variable digital literacy levels) to scaling health ambassador training efforts	Innovative methodologies were adopted to leverage technology.
Limited manpower for RCCE efforts.	Volunteers were actively recruited via schools and social media. Funds were raised to recruit staff.
Burnout and high turnover among volunteers.	Active training, engagement, appreciation, and refreshing of volunteers were done.
Face-to-face engagements were time-consuming and manpower-intensive.	Health engagement messages were curated into audio, video, and comic format and disseminated via loudhailers, social media, and text messaging channels.
Health Messaging	
Addressing real concerns accurately.	Migrant worker feedback about concerns and myths were obtained through face-to-face engagements, text messaging, and at medical posts. Responses were created after broad consultation with government departments, health experts, and pilot groups of migrant workers.
Difficulties in translations and proofreading of health messages.	Translators were recruited. Migrant workers assisted in proofreading. Standard operating procedures were established to streamline processes.
Limited capacity to distribute resources (e.g., print companies in lockdown, bureaucratic procurement processes, and dormitory managers overwhelmed by operational duties)	Processes were adapted to bypass institutional procurement processes and alternate dissemination pathways were quickly implemented.
Different facilities required different, tailored messages.	Facilities with similar challenges could share resources and others were tailored as needed.
Largely unstandardized RCCE efforts across facilities.	Resources developed were posted centrally on a website and shared nationwide to avoid duplication of efforts. A centralized RCCE team was developed to engage government authorities and migrant worker organizations to align efforts.
Working with different nonhealth sectors with different chains of command and outbreak experience.	Strong interpersonal relationships and trust had to be developed in the field. “MyBrotherSG” evolved as a networking platform for migrant worker organizations and various stakeholders, with a strong ethos of inclusivity, collaboration and noncompetitiveness.

*Note.* RCCE = risk communication and community engagement.

In August 2020, dormitories were declared cleared of the SARS-CoV-2 virus (Ministry of Manpower, 2021d, 2021e). At this point, the RCCE project gained attention from World Health Organization (WHO), a United Nations agency responsible for international public health, which granted the team a US$196, 000 grant to formalize their RCCE toolkit for scalability in the region. The team thus moved into a program consolidation phase where processes and structures were reviewed and feedback was shared at local steering committee meetings, international technical advisory group consultations, focus group discussions (FGDs) and key informant interviews with migrant workers. Consolidating the foundation of the program was integral for wider scale-up nationally. Discussions were analyzed qualitatively and used to inform a working logic model of the program. A theory of change that emerged is that increased levels of participation and engagement in RCCE activities among migrant workers will lead to the community’s increased sense of empowerment and autonomy, ability to prevent disease, and result in reduction in transmission of COVID-19 and improvement in overall health outcomes. Co-developing a theory of change enabled identification and assessment of key indicators to adapt program activities and maximize outcomes.

Reflection via stakeholder analysis highlighted key activities involved in establishing and implementing the RCCE program, including the tailored provision of information products, setting up an RCCE team comprising volunteers and staff, capacity building through training migrant worker ambassadors and mobilisers, governance through regular meetings, ongoing two-way dialogues with migrant workers, and research.

## Relevant Changes

The RCCE service has evolved to become “MyBrotherSG,” which offers a centralized networking platform bringing government authorities, health institutions, nonprofit organizations, and migrant worker representatives together monthly to align goals for maximal synergy in RCCE. As of September 2021, it has grown from fragmented efforts of individuals and organizations to an 18-partner network with four staff and 132 volunteers and a governance framework. Resources are hosted centrally on www.mybrother.sg. During this time, many outputs and outcomes were achieved.

Funding of US$332,000 from benevolent organizations allowed for print products (200,000 health booklets and 25,000 posters), digital products (158 videos, 28 comics, nine webinars, over 2,000 digital resource downloads), personal engagements (510 face-to-face engagements, 14 workshops, 12 on-ground outreaches) overseen by nine local steering committee meetings, and four technical advisory group meetings.

Additional metrics measured included an increased following of the “MyBrotherSG” social media page from 2,822 in November 2020 to 28,700 in September 2021 and an increased social media reach and engagements of 774,033 and 63,056 at their peaks, respectively. Live webinars reached 21,236 views per episode on average. An online survey with 750 workers conducted between December 2020 and February 2021 showed increased numbers of workers receiving sufficient, culturally competent health information in their own language regularly, *t*(748) = 2.09, *p* = .04 and *t*(748) = 2.99, *p* = .003, with webinars and comics being the most effective products in achieving these outcomes. There was an increase in self-reported feelings of empowerment and agency, *t*(748) = 3.04, *p* = .002 and *t*(748) = 4.12, *p* = .00, respectively. Evidence from nine FGDs with 48 workers in Bengali, Tamil, Burmese, and Mandarin languages reinforced these findings.

In the future, improvement in the quality of RCCE can be measured by proxy via development of standard operating procedures that guide resource development; shorter turnaround times; increase in collaborative, multi-agency projects through sharing of communication campaign calendars between partners; increase in funding for RCCE research and programs; and heightened awareness of RCCE as a response pillar among government ministries.

Currently, 90% of migrant workers are vaccinated and undergo weekly routine rostered testing to ensure quick containment of cluster outbreaks, and as of September 2021, cases in dormitories are lower than that in the community, with expectations to ease movement restrictions in dormitories (Ministry of Manpower, 2021a).

## Lessons Learnt

The effective delivery of scientific information through RCCE at the outset of a pandemic was limited by infrastructure, manpower, resources, and a lack of RCCE expertise. In spite of experiencing high levels of uncertainty, a firm commitment to delivering RCCE through leadership and governance structures, garnering senior stakeholder support for bottom-up RCCE efforts led by volunteers and drawing upon strengths within affected communities through participatory approaches to mobilize peer-led support and including community leaders into policy decision-making provided an enabling environment for effective scientific information dissemination with diverse groups. A key transition point in the scale-up of the program was shifting from a top-down, unilateral to human-centric participatory approach, where community leaders’ feedback became part of a regular two-way dialogue between affected communities and high-level policymakers, taking cultural and structural contexts of migrant workers into consideration (Sastry et al., 2021). Embedding data collection via surveys, FGDs, and key informant interviews proved key to adapting RCCE programs and adjusting policies to ensure relevance. Subsequent RCCE activities were adjusted to ensure continual, intentional community engagement with migrant workers to understand their contextual needs and ensure feedback was relayed to relevant authorities. The shift in RCCE approach was crucial for trust building, community empowerment, and effective scientific communication (Cyril et al., 2015).

Our experience reinforces well-articulated principles of optimizing the success of the RCCE program, including the following:

1. Early, proactive, and broad engagement of high-level stakeholders and policymakers to provide an enabling environment for bottom-up initiatives, to ensure national coordination and consistent reliable scientific information in rapidly changing situations;
2. Early and regular two-way engagement between policymakers and representatives of affected migrant worker communities;
3. Shift from unilateral to participatory practice approaches with a focus on agency, autonomy, and empowerment;
4. Use of data aligning with the RCCE global strategy;
5. Use of multiple modes of message dissemination; and
6. Commitment to setting up of governance structures, leadership, and scaling up.

Our experience shows that even in crisis settings naive to RCCE concepts, systems and structures can be developed responsively to produce adapted, consistent, coordinated, accurate, and timely RCCE in outbreak responses to ensure effective scientific communication for optimal results.
